# Less is more: Antibiotics at the beginning of life

**DOI:** 10.1038/s41467-023-38156-7

**Published:** 2023-04-27

**Authors:** Martin Stocker, Claus Klingenberg, Lars Navér, Viveka Nordberg, Alberto Berardi, Salhab el Helou, Gerhard Fusch, Joseph M. Bliss, Dirk Lehnick, Varvara Dimopoulou, Nicholas Guerina, Joanna Seliga-Siwecka, Pierre Maton, Donatienne Lagae, Judit Mari, Jan Janota, Philipp K. A. Agyeman, Riccardo Pfister, Giuseppe Latorre, Gianfranco Maffei, Nichola Laforgia, Enikő Mózes, Ketil Størdal, Tobias Strunk, Eric Giannoni

**Affiliations:** 1 Department of Pediatrics, Children’s Hospital Lucerne, Lucerne, Switzerland; 2 Paediatric Research Group, Faculty of Health Sciences, UiT-The Arctic University of Norway, Tromsø, Norway; 3 Dept. of Pediatrics and Adolescence Medicine, University Hospital of North Norway, Tromsø, Norway; 4 Department of Neonatology, Karolinska University Hospital, Stockholm, Sweden; 5 Department of Clinical Science, Intervention and Technology (CLINTEC), Karolinska Institutet, Stockholm, Sweden; 6 Neonatal Intensive Care Unit, Mother and Child Department, Policlinico University Hospital, Modena, Italy; 7 Division of Neonatology, Department of Pediatrics, McMaster Children’s Hospital, McMaster University, Hamilton Health Sciences, Hamilton, Canada; 8 Department of Pediatrics, Women & Infants Hospital of Rhode Island, Warren Alpert Medical School of Brown University, Richmond, USA; 9 Biostatistics and Methodology, CTU-CS, Department of Health Sciences and Medicine, University of Lucerne, Luzern, Switzerland; 10 Clinic of Neonatology, Department Mother-Woman-Child, Lausanne University Hospital and University of Lausanne, Lausanne, Switzerland; 11 Department of Neonatology and Neonatal Intensive Care, Medical University of Warsaw, Warszawa, Poland; 12 Service néonatal, Clinique CHC-Montlegia, groupe santé CHC, Liège, Belgium; 13 Neonatology and Neonatal Intensive Care Unit, CHIREC-Delta Hospital, Brussels, Belgium; 14 Department of Paediatrics, University of Szeged, Szeged, Hungary; 15 Neonatal Unit, Department of Obstetrics and Gynecology, Motol University Hospital Prague, Prague, Czech Republic; 16 Department of Neonatology, Thomayer University Hospital Prague, Prague, Czech Republic; 17 Department of Pediatrics, Inselspital, Bern University Hospital, University of Bern, Bern, Switzerland; 18 Neonatology and Paediatric Intensive Care Unit, Geneva University Hospitals and Geneva University, Geneva, Switzerland; 19 Neonatology and Neonatal Intensive Care Unit, Ecclesiastical General Hospital F. Miulli, Acquaviva delle Fonti, Italy; 20 Neonatology and Neonatal Intensive Care Unit, Policlinico Riuniti Foggia, Foggia, Italy; 21 Neonatologia e Terapia Intensiva Neonatale, University of Bari, Bari, Italy; 22 Perinatal Intensive Care Unit, Department of Obstetrics and Gynaecology, Semmelweis University, Budapest, Hungary; 23 Institute of Clinical Medicine, University of Oslo and Oslo University Hospital, Oslo, Norway; 24 Neonatal Directorate, Child and Adolescent Health Service, King Edward Memorial Hospital, Perth, Western Australia

**Keywords:** Health care, Paediatrics, Neonatal sepsis

## Abstract

Antibiotic exposure at the beginning of life can lead to increased antimicrobial resistance and perturbations of the developing microbiome. Early-life microbiome disruption increases the risks of developing chronic diseases later in life. Fear of missing evolving neonatal sepsis is the key driver for antibiotic overtreatment early in life. Bias (a systemic deviation towards overtreatment) and noise (a random scatter) affect the decision-making process. In this perspective, we advocate for a factual approach quantifying the burden of treatment in relation to the burden of disease balancing antimicrobial stewardship and effective sepsis management.

## Abstract (web summary)

Fear of missing neonatal sepsis has led to early in life antibiotic administration, even without culture-proven sepsis. Here, the authors discuss the potential impact on antimicrobial resistance, and chronic disease later in life, due to effect on the developing microbiome, suggesting a factual based approach in quantifying burden of treatment in relation to the burden of disease.

## Introduction

Physicians caring for neonates presenting with respiratory distress or other nonspecific symptoms often prescribe empiric antibiotics because early-onset sepsis (EOS) cannot be ruled out. The rationale behind this practice is the possible risk of infection and the potential lifesaving effect of early initiation of antibiotics. Serious bacterial infections lead to the death of around 400,000 newborns worldwide each year and puts survivors at risk of lifelong disability^1,2^. Antibiotic therapy is one of the key achievements in medicine in the last century, reducing infection-related morbidity and mortality^3^. For neonates with culture-proven sepsis, prompt initiation of antibiotic treatment is undoubtedly lifesaving^4^. However, the potential involvement of bacterial infections in many clinical syndromes, together with the limited accuracy of current sepsis diagnostic tests resulted in a massive use of antibiotics^5^. After implementation of *Group B streptococcal* sepsis prevention strategies in the 1990s, the incidence of culture-proven EOS has substantially decreased in high-income countries, and is continuing to go down in the new millennium^6–8^. Mortality of neonatal sepsis has similarly decreased in high-income countries^9^. Despite these positive changes, antibiotics are still the most commonly prescribed medication during the first days of life^6,9^. Up to 14% of late-preterm and term neonates and up to 90% of extremely preterm neonates receive intravenous antibiotics^10^. This exposure is disproportionate to the burden of disease, as only 0.5 to 2% of neonates treated with antibiotics have a culture-proven bacterial infection^11^.

During the last decades, the impact of misuse and overuse of antibiotic therapy on growing antimicrobial resistance (AMR) has been increasingly acknowledged as a global public health threat. The World Health Organization (WHO) has called for urgent action to improve antibiotic treatment by increased awareness and evidence through education, surveillance and research to avoid AMR crisis^12^. Whereas the impact of antibiotic use on AMR is well known, evidence as to the effect of antibiotic therapy on the developing microbiome is relatively new and dynamic^13^. This is particularly important at the beginning of life, where the commonly prescribed course of 48 h of empiric antibiotic therapy for suspected sepsis in term infants may exert major effects on the microbiome and AMR gene selection, which remain detectable at one year^14,15^. In preterm infants, unwarranted and prolonged antibiotic exposure within the first few weeks of life may increase the subsequent risk of necrotizing enterocolitis, bronchopulmonary dysplasia, late-onset sepsis and death. Some studies, with an inherent bias due to the retrospective design, reported conflicting results; reinforcing the need for well-powered, prospective studies to clarify the causal relationship and the effect-size^16–20^. Further, early life antibiotic exposure is associated with future health problems like obesity, allergic predisposition, asthma, diabetes, juvenile idiopathic arthritis, celiac and inflammatory bowel disease^14,21,22^. Whereas causal associations between early exposure to antibiotics, changes in the microbiome and diseases presenting later in life are not fully understood or analysed, growing evidence from the current literature underlines the interplay between the microbiome and genomics, proteomics and metabolomics. Various mechanisms within the gut-brain, gut-lung, and gut-skin axis, and immune modulation are reported to contribute to the pathogenesis for diseases presenting later in life^23–30^. The example of microbe-induced obesity may serve as proof of concept: In a mouse-model, Cox et al. were able to proof the causal role of penicillin-altered microbiome for long-term metabolic changes and obesity^31^. Therefore, lowering exposure to antibiotics at the beginning of life may reduce the risk for future chronic health conditions. That is why less antibiotics at the beginning of life is urgently needed.

In this perspective, we review the current state of antibiotic exposure at the beginning of life and the incidence and outcome of culture-proven EOS. We hypothesize that a factual approach quantifying the burden of treatment in relation to the burden of disease may help balancing antibiotic stewardship and efficient sepsis management.

## The current state

There is an inappropriate antibiotic exposure in relation to the number of culture-proven infections at the beginning of life with a potentially high societal cost of treatment due to AMR-development and perturbations of the developing microbiome^13,32–35^. In 2019, an estimated 5 million people worldwide died from illnesses in which bacterial AMR played a part, including 1.27 million deaths directly caused by drug resistant infections^13^. The number of people living with chronic conditions potentially triggered by early-life microbiome disruption is increasing, leading to substantial mortality and morbidity^36^. Obesity and diabetes mellitus show an exponential rise within the last decades, and are associated with subsequent mortality due to cardiovascular disease^37^. Globally, the prevalence of asthma has increased to 11.5% in the population aged between 5 and 69 years^38,39^. Inflammatory bowel disease (IBD) has a high impact on individual health and on healthcare systems. In the 21^st^ century, IBD has become a global disease with a prevalence of 0.3% in Europe and North America and its incidence is rising in newly industrialized countries from Asia, South America and Africa^40^. As preventive interventions have a limited success rate, there is an urgent call for new strategies^41^. Preservation of a healthy microbiome through a more rational use of antibiotics is an important point of action to prevent numerous diseases and improve global health^42–45^.

**Fig. 1:**
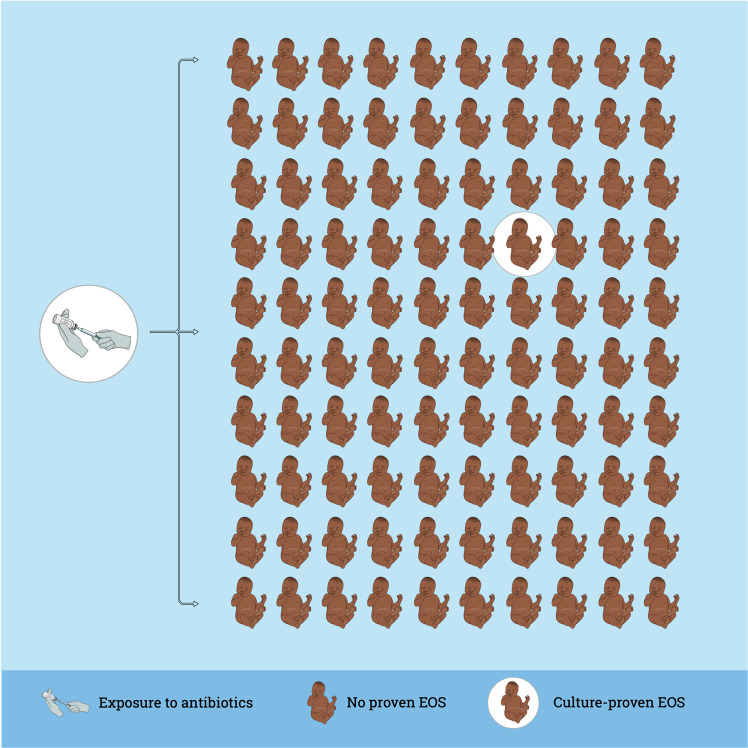
Overtreatment with antibiotics at the beginning of life. The rate of antibiotic exposure at the beginning of life of life varies between 1.2 and 14%^11,32,47–49^. The incidence of culture-proven early-onset sepsis (EOS) varies between 0.13 and 1.45/1000 term and late-preterm neonates^11,32,53–57^. Therefore, between 50 to more than 100 term and late-preterm neonates are started on antibiotics for each case of culture-proven EOS.

The burden of EOS in high-income countries is decreasing within the new millennium, as the incidence of EOS and sepsis-related mortality are declining in term and late-preterm neonates^7^. Important to note, the situation in low-income countries may differ due to limitations in access to health care and much higher sepsis-related mortality^46^. The recently published AENEAS study (Antibiotic Exposure for Suspected Neonatal Early-onset Sepsis) shows the current situation in 13 different networks from Europe, North America, and Australia^11^. In a cohort of 757,979 late-preterm and term infants, 21,703 (2.9%) infants received intravenous antibiotics during the first postnatal week. This proportion is in the lower range compared to the literature with reports up to 14% all infants treated^32,47–49^. However, wide practice variation from 1.2% to 12.5% of all infants started on antibiotics was observed among different networks^11^. Infants with culture-proven EOS were treated for a median of 9 (IQR 7–14) days and those who were started on antibiotics but did not have a positive blood culture were treated for a median of 4 (IQR 3–6) days, in line with the recent literature, but in contrast to international guidelines requesting to discontinue antibiotics after 36 to 48 h^50–52^. Within the AENEAS cohort, the incidence of culture proven EOS was 0.49/1000 live births (range 0.18–1.45) and EOS-associated case fatality was 3.2%^11^. In the recent literature, EOS rates between 0.13 and 0.95/1000 term and late-preterm live births were reported in Europe, the United States and Australia^32,53–57^. Reports from these areas show a low mortality due to EOS and are in line with the AENEAS study results^32,50,53,58^. Between 50 to more than 100 term and late-preterm neonates are therefore started on antibiotics for each case of EOS (Fig. 1). Thus, antibiotic exposure at the start of life is very high, and the risks and cost of treatment are inappropriate compared to the burden of disease. Therefore, a new balance between effective sepsis care and antimicrobial stewardship (AMS) is urgently needed.

A number of today’s pediatricians and neonatologists were trained in an era with a prevailing mind-set that it is better to treat every neonate with clinical signs possibly related to bacterial infection, even if the risk of sepsis is low. Shifting this paradigm requires a culture change^59^. Initiating and implementing change successfully first requires an understanding of why change is necessary. The 8-steps-framework for leading change by John Kotter requests to create a sense of urgency as a mandatory first step, which was successfully applied in different healthcare setting^60,61^. The current overexposure of antibiotics at the beginning of life and its unintended consequences are why we must change.

## The needed change: A factual approach

Given that more than 98% of neonates started on antibiotics do not turn out to have a microbiologically documented infection, there is enormous potential for improving antibiotic prescription practices^11,32^. Currently, guidelines to decide when to initiate antibiotics in the first days of life show wide variations among countries^62^. There is no obvious single best strategy to safely reduce neonatal antibiotic exposure at the beginning of life and improve the balance of efficient sepsis care and AMS^11^.

**Fig. 2:**
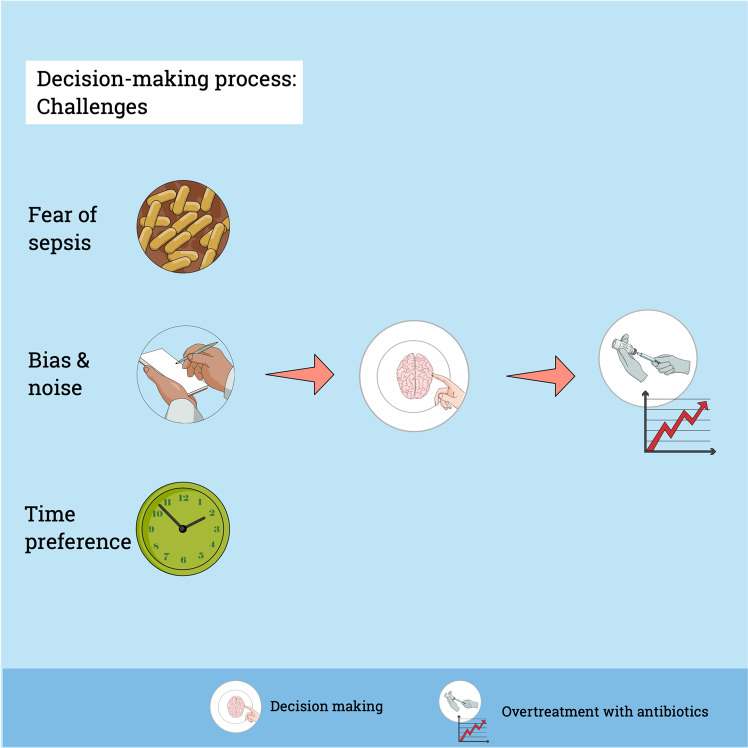
Challenges of decision-making. Fear to miss a true sepsis case and the impact of bias and noise on decision-making are important factors contributing to antibiotic overtreatment. In addition, there is a time preference for now with a discount over time for potential late benefits: the perceived immediate safety of antibiotic treatment for suspected sepsis is of much higher value than the possible decrease in risk for chronic diseases years or decades later^71^. These challenges of decision-making result in overtreatment with antibiotics at the beginning of life.

AMS is a key topic in current neonatal care and many clinicians are focused on improving antibiotic prescription practices. Nevertheless, safety concerns, fear of medical malpractice litigation, peer pressure and the tendency not to change antibiotic treatments started by colleagues commonly overrule AMS intentions^63^. Fear to miss a sepsis case is a key driver for antibiotic prescriptions in medicine^64^. The perceived vulnerability of children and seeking safety in the face of uncertainty are two important aspects guiding the decision to start antibiotics in paediatric care (Fig. 2)^63,65^. A qualitative study in paediatric intensive care regarding decision making for antibiotic therapy observed an overruling of the clinical reasoning process by disease severity and safety concerns^63^. Importantly, possible adverse effects of antibiotics play a minor role in the prescribing process^66^. Fear however is a poor advisor for decision making and we have to calculate the risk to control the fear instinct^67^. A factual approach using data regarding the absolute risk of EOS and sepsis-related adverse outcomes may help to overcome the fear instinct and to improve the balance between efficient sepsis care and AMS.

Whereas fear can induce a bias with systematic deviation towards increased antibiotics use, many other factors impact neonatal antibiotic prescription. In addition to objective differences between guidelines, patient populations and implemented AMS programs, it is difficult to assess the impact of habits, collaboration across hierarchy and previous experiences^68–70^. The theory of time preference of behaviour and economic scholarship offers another explanation on how physicians trade off time and outcomes: there is a time preference for now with a discount over time for potential late benefits^71^. In this context, the perceived immediate safety of antibiotic treatment for suspected sepsis is of much higher value than the possible decrease in risk for chronic diseases years or decades later (Fig. 2). Nevertheless, with a factual approach illustrating the true risk, the bias from time preference may be reduced^72^. In addition, physicians make different decisions in similar situation at different points in time^73^. This phenomenon is described as noise, resulting in a random scatter of decision making (Fig. 2)^74^.

Physicians caring for neonates with nonspecific clinical signs such as respiratory distress often prescribe antibiotics due to their concern that the patient may have bacterial sepsis. This judgment is only verifiable if the blood cultures become positive. Otherwise, the quality of the decision remains blurred and only the quality of the decision-making process can be assessed^74^. However, this seldom happens and an inadequate circle based on causal thinking, confirmation and desirability bias may lead to overconfidence of physicians (Box 1). Learning from error is difficult. Failure can trigger a threatening feeling and these emotions may hinder learning^75^. An important step for every critical review and to enter a circle of learning (Box 1) is to use measures of decision hygiene^74^. The most important measure of decision hygiene is to take an outside view and to assess the baseline-rate of events. As for the fear instinct, a factual approach may help to improve the decision-making process by taking the outside perspective analysing baseline-rates and outcomes. Different baseline-rates and outcomes in different settings may further highlight the importance of using a factual approach.

Box 1. Cycle of overconfidence and cycle of learning A physician starts antibiotic treatment in a baby with clinical signs of respiratory distress compatible with retained lung fluid and other non-infectious diseases. The day after, the blood culture shows growth of *group B streptococci*. Without analysis of the decision-making process, the physician will most likely come to the conclusion, that gut feeling or experience were responsible for the correct decision to start antibiotics. Gut feeling may be translated as knowing without knowing why. Experience is the result of causal thinking and both will increase the confidence of the physician^74^. But the statistical evidence is completely different: even experienced neonatologists most often start antibiotics without later confirmation of culture-proven early-onset sepsis (EOS). If the blood culture of the described baby remained negative, the physician may still conclude that starting antibiotics was the right decision due to the possibility of culture-negative EOS. But culture-negative EOS is a controversial diagnosis, without clear diagnostic criteria and there is a high risk that as physicians we may diagnose a culture-negative sepsis by conclusion on a selective and distorted evidence due to the known conclusion bias, which is mainly dependent on the desire to confirm our past decision (desirability and confirmation bias)^74^. If there is obviously no sepsis, the physician may stop the unnecessary antibiotics without review of the decision due to subjective ignorance. Causal thinking, outcome and confirmation bias, and subjective ignorance may lead to a cycle of overconfidence.

On the other hand, if the decision to start antibiotics was correct and the blood culture becomes positive, the physician may analyse the decision-making approach for learning. A safety-II-approach, learning by enhancing the adaptive capacity, is a possibility to learn from positive experiences within the system compared to the traditional linear learning from errors (safety-I-approach)^99,100^. A critical review of the correct decision may give new learning points and findings for the future. The willingness to consider alternatives already creates new knowledge. If the blood culture remains negative, after a critical review of the situation the physician may end up with the diagnosis of culture-negative sepsis, but probably less often if we successfully avoid confirmation bias^35,70^. To take an outside view and to assess the baseline-rate of events is the most important measure of decision hygiene^74^. If there is no sepsis and antibiotics were unnecessary, the physician may critically review the decision and conclude that the future course was not predictable (objective ignorance)^74^.

## The first steps towards a factual approach

**Fig. 3:**
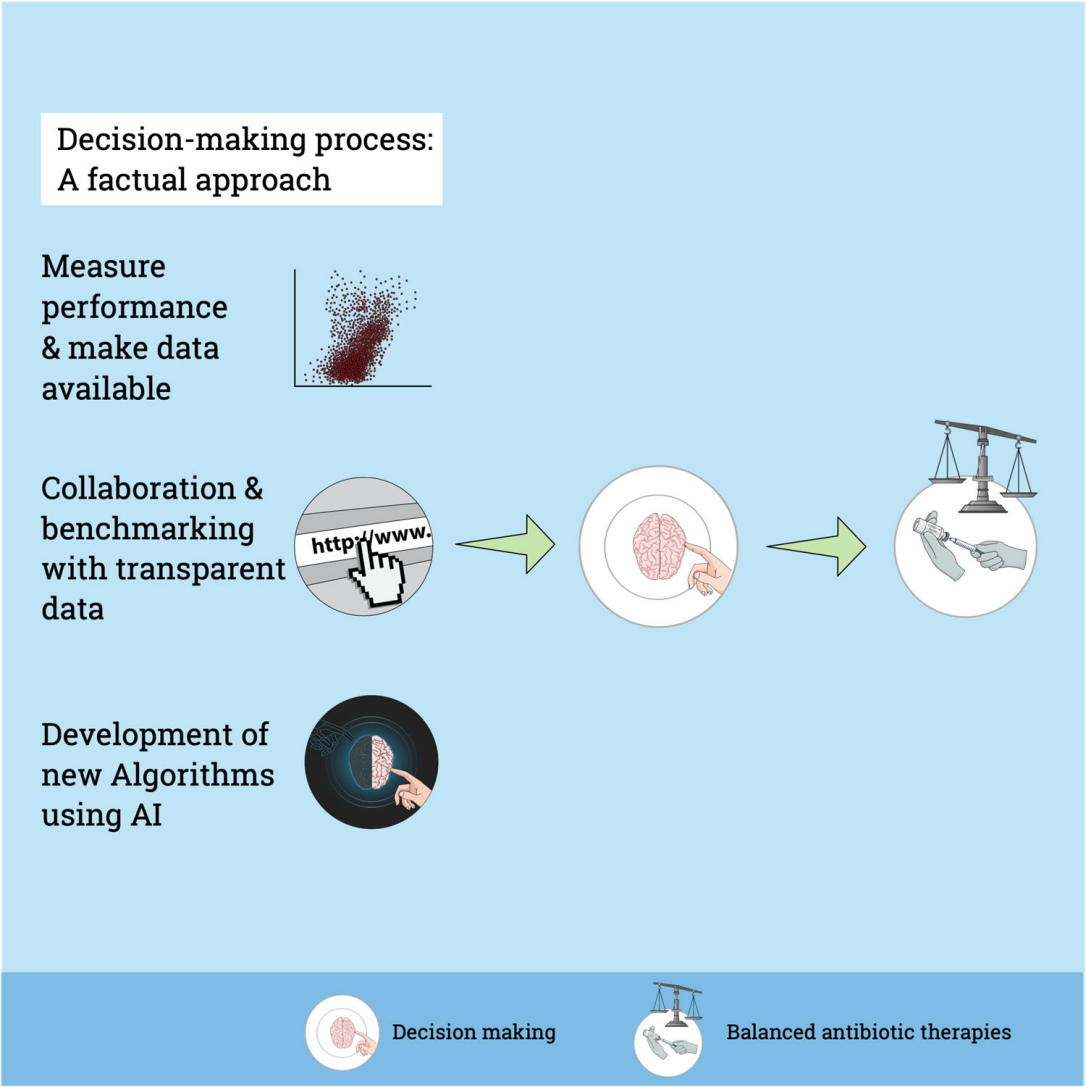
A factual approach for decision-making. The first step towards a factual approach is to measure one’s own performance and to make data available within the institution. International, open-access data presentation may help for collaboration and benchmarking improving learning and decision-making. In future, algorithms and artificial intelligence will play an increasing role for individualized decision-making, taking into account routine data and new variables. In the end, a factual approach may help to balance effective sepsis care and antimicrobial stewardship.

In discussions with neonatologists, antibiotic prescriptions are often not acknowledged as a problem. This statement is rarely supported by data. To think we are doing well, is not good enough—we must know. The first step towards a factual approach is to measure one’s own performance (Fig. 3). Measuring performance is a mandatory step to get better^76^. Auditing routinely available outcome data is becoming more common and may help to overcome this gap. Importantly, weaknesses and shortcomings in routine data research is a cause of concern and must be addressed^77^. A recently published WHO review regarding the use of digital health technologies reported an increasing problem of global inequity^78^. Transparent and open-access data presentation is key to minimize inequity. Therefore, we must measure our own performance and make these data available to all physicians involved in care in every institution.

The COVID-19 pandemic showed us the potential benefit of international collaboration and transparent data sharing. Timely and accurate presentation of data with dashboards was the base for benchmarking and collaborative learning. The team of the Johns Hopkins University in Baltimore reported several challenges with their collected data, such as ambiguous and inconsistent parameter definition, and lack of reporting standardization including support for machine-readable data and unstable reporting practices in terms of metrics and frequency^79^. Lessons learnt from scientific data presentation include freely accessible results as standardised graphs and figures with a high grade of simplicity and clarity, attention to concise titles and subtitles, regular feedback of users to facilitate the education of people and ensuring trust by full transparency^80^. In addition, the authors conclude that the positive experience of the COVID-19-dashboard may be transferred to other topics or other medical diseases^80^.

The conduct of the AENEAS study allowed us to learn important lessons to take the next step in our endeavour to reduce antibiotic use at start of life without affecting sepsis-related outcomes and to improve the balance between efficient sepsis care and AMS^11^. Feasibility, high data quality and insightful data presentation were key factors. Therefore, most important for feasibility and high data quality is the definition of a standardized, minimal data set with concise definitions of parameters. The AENEAS study may serve as a pilot trial. With only 6 key parameters we were able to analyse the burden of disease (1. incidence of EOS; 2. sepsis-related mortality), the burden of antibiotic treatment (3. proportion of live births started on antibiotics; 4. duration of antibiotic treatment) and a baseline description of the cohort (5. gestational age; 6. all-cause neonatal mortality)^11^. The timely, transparent display of data within a dashboard is the main difference from more conventional registries. Data of registries are often only accessible after being published and transparency is limited to participating centres^81^. In conclusion, international collaboration and benchmarking of open-accessible data may leverage learning and decision-making to improve care at the beginning of life.

Due to the low incidence of culture-proven EOS and sepsis-related mortality, randomized clinical trials (RCT) require a very large study population to prove the safety of an approach^51^. In addition, RCTs in neonatal sepsis are challenging and not always feasible due to high ethical requirements, difficulties to obtain parental consent during an emotional time period, and high complexities and costs. Nevertheless, the recently published REASON trial demonstrates that a RCT evaluating the effect of antibiotics on the microbiome, metabolome and immune markers at the beginning of life is feasible^82,83^. On the other hand, new clinical trial designs using routine data from the real world may help to overcome these problems^84^. The aim is to develop a tight link between clinical care and research. Therefore, we need a change in the mind-set of many care providers: On one hand, as physicians, we are responsible to deliver the best possible care. On the other hand, clinical research is mandatory to define best possible care. The new stream of clinical research, using routine data from the real world, necessitates that physicians and other health care workers act as care providers and as clinical researchers: To deliver the best possible care and to strive to get better^84^. Therefore, the development of new algorithms including artificial intelligence analysing routine data may help to reach the next level of a factual approach. In the future, data-based individualization including results from multiomics analyses may further improve the accuracy of prediction^85–87^.

## Limitations and next steps

The strengths of the AENEAS six-key point dashboard to consolidate a factual approach lies in the feasibility and standardized data including two dimensions; the burden of disease and the burden of treatment^11^. The presentation of both dimensions is mandatory to balance efficient sepsis care and AMS. However, such a dashboard also has limitations. First, AENEAS was limited to late-preterm and term neonates with suspected EOS. Antibiotic exposure of more immature preterm infants with suspected EOS and late-onset sepsis (LOS) needs to be evaluated as well and is achievable, with sufficient resources. Whereas very preterm infants account for less than 2% of life births, the rate of EOS (13.5/1000 very preterm infants) and LOS (93/1000 very preterm infants) are significantly higher^88–90^. Secondly, due to the minimal data set, more in-depth information regarding morbidity of EOS and management strategies are lacking. With a more detailed data sampling in a subset of patient cohorts and centres, we may mitigate these limitations. Analyses of more detailed and richer data sets may reveal currently unappreciated risk profiles affecting antibiotic treatment thresholds and management strategies. In addition, there is a need for regular quality improvement cycles after start of the initiative and the initial data set may be changed accordingly.

Most importantly, a dashboard showing the burden both of confirmed EOS cases as well as antibiotic exposure is not the final aim. Registry data are rarely used to evaluate the impact of therapeutic interventions on patient outcomes^81^. To allow collaborative learning and to promote change, longitudinal presentation of standardized key indicators and transparency are mandatory^91,92^. Longitudinal (inter)national benchmarking allows clinicians to assess their own performance and to guide improvements^93^. The development of adjustable benchmarks may facilitate global use in diverse settings. Insightful data presentation showing the balance between the burden of EOS and the burden of antibiotic treatment may help to guide clinicians regarding future steps to improve their performance and to foster collaborative learning. Among many challenges ahead, the most urgent is to develop better diagnostic tools for neonatal sepsis. Non-bacterial infections can also cause sepsis, and the lack of a clear definition impedes our studies and efforts to improve the management of neonatal sepsis^94^. A recent review demonstrated the highly variable sepsis definitions used even in clinical trials^95^. Whereas adult and pediatric sepsis definitions mandate evidence of organ dysfunctions, neonatal sepsis definitions are generally restricted to clinical signs, microbiology, and laboratory results, and intention to antibiotic treatment.

Culture-negative sepsis represents another common, poorly defined and challenging condition. The AENEAS study reported only on culture-positive EOS. There is no consensus definition of culture-negative sepsis and in literature, the rate of culture-negative sepsis is reported to be 6–16 times higher than culture-positive sepsis^70^. Neonatal, culture-negative sepsis exists, but non-infectious conditions that mimic sepsis are far more common and include prematurity, transient tachypnea of the neonate, surfactant deficiency, persistent pulmonary hypertension and many more. Fear of true culture-negative sepsis is a driver for prolonged antibiotic therapy. Optimal blood culture technique and intensified diagnostic search for non-bacterial sepsis-like causes are key in every neonate with suspected EOS. In their recently published framework, Cantey et al. used a factual approach to tackle the overdiagnosis of culture-negative sepsis: They calculated the risk of culture-negative sepsis based on the incidence of sepsis, the probability of bacterial detection in a culture, and the risk of bacterial ultra-low concentration below the detection threshold of cultures with a need of antibiotic treatment. They concluded that even with conservative assumption, the rate of culture-negative sepsis should be 8 to 10 times lower than the rate of culture-positive sepsis^35^. This is around 100 times lower than observed in the literature^11,70^. New and improved blood culture techniques, viral and bacterial detection with PCR panels, and omics techniques that can detect host response to microbial invasion are potentially beneficial techniques when considering the differential diagnosis of culture-negative sepsis. Thus, rule-out sepsis is not a diagnosis, and culture-negative sepsis is most likely often a wrong diagnosis.

In the recent literature, the EOS-calculator has been the most discussed approach to reduce antibiotic therapy for suspected EOS in late preterm and term infants^48^. The EOS-calculator is a fact-based model including the local incidence of EOS, maternal risk factors and neonatal clinical signs resulting in management advice. In settings of high exposure, studies using the EOS-calculator report a substantial reduction in the use of antibiotics in the first week of life^48^. The impact of the EOS-calculator in settings with a strong culture of AMS is not studied and therefore debatable. The risk threshold of 3 EOS-cases out of 1000 as an indication to start antibiotics may be a possible reason for the questionable benefit of the EOS-calculator in settings with relatively low antibiotic use^48^. Moreover, the EOS-calculator does not identify all cases of EOS, but this is also the case with other approaches^96^. Interestingly, accepting an algorithm only until an error or limitation is found is a general known barrier in the implementation of algorithms for decision making, even if reports prove the overall superiority of the algorithm^74^. The AENEAS study reported networks with a substantially lower antibiotic use as reported with the use of the EOS-calculator. A strategy of serial physical examination has been associated with markedly lower antibiotic exposure than in networks using the EOS-calculator, but more prospective studies are needed^97^.

As we know from the management literature, there is no lack of good ideas in the world, but a lack of implemented good ideas^98^. The AENEAS study was a start to show the feasibility and possible analyses for a factual approach. An open-access data dashboard including international data from high-, middle- and low-income countries may help to leverage collaborative learning and new thinking within an initiative aiming to conciliate efficient sepsis care and AMS.

## Conclusions

Antibiotic exposure at the start of life is high and the risks and costs of treatment are disproportionate compared to the burden of EOS. Fear to miss a true sepsis case, the impact of bias, noise, and time preference on decision-making are important factors contributing to antibiotic overtreatment. We hypothesize that the decision-making process regarding antibiotic therapy at the beginning of life may be improved with a factual approach. First, we must measure one’s own performance and make these data available. This feedback may produce a sense of urgency regarding the needed change of behaviour. Secondly, collaboration and benchmarking using an open-access dashboard quantifying the burden of disease versus the burden of treatment may be an important step to initiate a global initiative to conciliate effective sepsis care and antimicrobial stewardship. In particular, the focus should be made on educational interventions to improve the decision-making process regarding the start and the stop of antibiotics. And thirdly, the development of new algorithms using artificial intelligence analysing routine data may leverage decision-making to the next level of a factual approach. The aim of a preserved microbiome and reduced antimicrobial resistance may improve the health of future generations.
